# Enhancing construction site performance through technology and management practices as material waste mitigation in the Malaysian construction industry

**DOI:** 10.1016/j.heliyon.2024.e28721

**Published:** 2024-03-28

**Authors:** Mahdi Mohammed Abdullah Abkar, Riduan Yunus, Yaser Gamil, Mohammed Abdo Albaom

**Affiliations:** a Faculty of Civil Engineering and Built Environment Universiti Tun Hussein Onn Malaysia (UTHM) Parit Raja, Batu Pahat, Johor, 86400, Malaysia; b Department of Civil Engineering, School of Engineering, Monash University Malaysia, Jalan Lagoon Selatan, 47500, Bandar Sunway, Selangor, Malaysia; c Department of Civil, Environmental and Natural Resources Engineering, Luleå University of Technology, Sweden; d Faculty of Computer Science and Information Technology, Department of Computer Science, Universiti Putra Malaysia, Serdang, 43400, Malaysia

**Keywords:** Technology and management approaches, Solid waste, Material waste mitigation, Site performance, Malaysian construction industry

## Abstract

The construction industry, increasingly prioritizing sustainability, necessitates an exploration of technology and management's role in mitigating material waste at construction sites. This study examines the impact of 3R, IBS, BIM, and MMA in enhancing Construction Site Performance (CSP) in the Malaysian construction sector. Seven hypotheses were formulated to assess the relationship between technology adoption, material management practices, and the moderating influence of Material Management Adoption (MMA) on CSP. Data were collected through an online survey from 295 valid responses in the Malaysian construction sector, focusing on professionals involved in solid waste management. Utilizing Partial Least Squares - Structural Equation Modeling (PLS-SEM) and Statistical Package for the Social Sciences (SPSS), the findings highlight the importance of technological integration, efficient material management, and competitive strategies in effective material waste mitigation. Furthermore, the qualitative aspect of the study, conducted among 6 solid waste organizations in Malaysia, enriches the findings by providing nuanced insights into local practices and challenges. Emphasizing the importance of contextual insights, the study addresses professionals involved in solid waste management within the Malaysian construction industry. The geographical specificity adds depth to the analysis, offering a comprehensive understanding of regional dynamics. Despite acknowledging limitations in technology and material usage, the study offers recommendations for refining waste mitigation and improving construction site performance. This research model offers actionable insights for construction site stakeholders, emphasizing the criticality of waste mitigation and CSP. The results, both quantitative and qualitative, underscore the potential of these practices within the Malaysian construction industry to foster innovation and drive positive change.

## 1 Introduction

The construction industry is a vital and influential economic sector, having a long history and significant size. It plays a crucial role in contributing to the nation's economy. However, it is also closely tied to environmental issues, mainly due to a lack of proper management and technology implementation for resource management in construction [1]. Construction technology employs sophisticated tools, equipment, and procedures to streamline tasks, diminish manual work, and boost effectiveness across construction operations. Its role encompasses waste reduction, resource optimization, and lessening environmental repercussions. This integration intertwines productivity, expenses, and technological advancements, fostering economic advancements and sectoral progress [2]. Material management practices have been recognized as essential for project delivery, quality, and cost-effectiveness in the construction industry. However, interconnected environmental challenges arising from inadequate resource management and technology implementation within the construction industry present a pressing engineering practice problem [3]. Malaysia's rapid development and urbanization have resulted in significant environmental impacts, with approximately 8 million tonnes of construction waste generated annually, affecting social and economic infrastructure [4]. Construction waste has become a significant concern, posing hazards to both nature and humans [5]. Construction waste management faces challenges due to factors such as waste generation, consumption habits, and population growth [5]. The construction sector is responsible for a significant portion of global environmental impact, consuming 40% of raw materials and generating nearly 50% of carbon emissions [6,7]. Over the past few years, numerous researchers have directed their attention toward advancements in waste sorting technology, particularly in the sorting stage. Some of these researchers have utilized artificial intelligence, big data, and other technologies to enhance the efficiency of waste sorting processes and elevate the quality of sorting outcomes [8].

In Malaysia, the construction waste generated during construction activities is estimated to reach approximately 30%–35% of the total project production, with projected costs reaching 368.31 tons per day by 2023 [9]. In addition, the absence of comprehensive government policies addressing construction waste management exacerbates issues related to inefficient resource utilization and environmental sustainability within the construction industry, presenting an additional challenge to be addressed [4]. This lack of policy exacerbates issues stemming from inadequate resource management and technology implementation, thereby impacting both operational efficiency and sustainability within the construction sector. Notably, Malaysia's rapid urbanization has intensified environmental concerns, resulting in alarming levels of construction waste generation [2].

Construction waste accounts for a substantial proportion of project production, emphasizing the need for waste reduction and recycling [8]. Concrete waste constitutes a significant portion of construction waste, highlighting the need for effective management strategies [10]. Illegal dumping sites and inadequate site management practices contribute to the construction waste problem [ [[3], [4], [5], [6], [9], [7], [8], [10], [11]]]. The adoption of the Reduce, Reuse, and Recycle (3R) approach and Industrialized Building Systems (IBS) can effectively reduce material waste and improve construction site performance [ [[8], [10], [11], [12]]]. The integration of Building Information Modeling (BIM) technology enhances project quality, reduces delays and costs, and facilitates data management and communication among stakeholders [13,14]. Robotics systems offer benefits in terms of construction productivity and performance [ [15,16]]. While adding to the body of knowledge and emphasizing the relevance of technology as material waste management, the study appropriately suggested such prospective strategies and recommendations for the advantages of technology innovations' uses toward material waste mitigation and construction site enhancement for future researchers and developers, with clear highlights for the theoretical, practical, and methodological consequences in the same context [17]. The absence of technology and material management strategic approaches during construction processes can lead to project delays, increased costs, and the accumulation of more waste on construction sites [17]. Therefore, this study aims to investigate the impact of technology adoption, specifically the incorporation of the Reduce, Reuse, and Recycle (3R) approach, Industrialized Building Systems (IBS), and Building Information Modeling (BIM), on material waste mitigation and Construction Site Performance (CSP) within the Malaysian construction sector. Additionally, the research seeks to assess the moderating role of Material Management Adoption (MMA) in influencing the relationship between technology adoption and CSP. Nevertheless, further research and enhancements in construction waste management practices, particularly in developing nations, remain imperative [18,19]. This investigation holds paramount importance in informing engineering practices and propelling the advancement of sustainable construction methodologies, especially in the context of developing nations where the need for efficient waste management practices is particularly pronounced as the sustainability in the context of construction waste entails the utilization of readily biodegradable construction materials and the recycling of construction waste within construction projects. [ [[16], [17], [18], [19], [20], [21]]]. The knowledge gap addressed in this research is the investigation of the association between technology and management practices and approaches for material waste mitigation towards construction site enhancement. Since there has been no extensive study into material waste mitigation with dimensions utilizing 3R, IBS, BIM as one direction for CSP, besides MMA as moderation in this relationship. Overall, the research findings underscore the significance of leveraging technological advancements for mitigating material waste, integrating strategies like the 3R approach, Industrialized Building Systems (IBS) technology, Building Information Modeling (BIM), and Material Management Adoption (MMA) to enhance construction site performance within the Malaysian construction industry.

## 2 Literature review

### 2.1 Factors affecting material waste mitigation at the construction site

As emerging nations put their technology in place during the conceptual design phase, the annual growth in several of these nations has increased highly in recent years, often surpassing the pace of expansion of the economy overall. A thorough explanation of the terminologies is required to understand the idea of robotics and how it relates to the building sector [22]. These nations sometimes use more efficient uses for their existing share of construction production and development, producing a booming sector that requires an efficient and unique strategy to boost the productivity and quality of work performed [23]. Malaysia is a developing country with limited knowledge and skills in the part of material management and its waste in fact, implementing such a proper waste management technology is the long-term solution for lowering construction costs, preventing the depletion of natural aggregate resources, resolving the landfill problem, and protecting the natural environment [[7], [8], [9], [10], [11], [12], [13], [14], [15], [16], [17], [18], [19], [20], [21], [24], [25], [26]]. Technologies are used to improve the efficiency and performance of buildings in various aspects of the construction industry [27]. As per [28], the construction's 3R approach typically delineates waste management actions for specific waste types. Additionally [29], underscores the overarching significance of 3R practices for all construction site stakeholders [30]. Moreover, the integration of Building Information Modeling (BIM) technology substantially influences material management within Malaysia's construction sphere, playing a pivotal role in industry advancement. BIM's implementation mitigates construction delays, minimizes additional expenses, and addresses communication challenges [15].

The Malaysian government's push for advanced technology like IBS aims to replace conventional construction methods [31]. Implementing IBS on construction sites enhances quality, safety, and cost-effectiveness while reducing material waste [25]. Despite this, challenges persist due to the reliance on foreign workers lacking expertise in IBS and the low adoption of these technologies in Malaysia's construction sector. Emerging material management technologies have potential in cost reduction and enhance stakeholder involvement [31] Recognizing these gaps, this research delves into technology and management approaches for waste mitigation to improve construction site performance in Malaysia.

#### 2.1.1 Material waste mitigation utilizing the reduce, reuse, and Recycle (3R) approach

Waste management poses a formidable challenge for both the contemporary era and the forthcoming generations, heralding an unfortunate future for those compelled to inhabit an environment marred by pollution. Every day in Malaysia, an excess of 23,000 metric tons of waste is generated [32]. In addition, the worldwide accumulation of metallic waste out of construction work presently stands at 1.3 billion tons, with projections indicating a staggering increase to 27 billion tons by the year 2050 [33]. Additionally, material waste reduction was seen as a method that improved the processes of project delivery and the quality, besides the effective cost of buildings that needed to meet the required standard and its obvious usages in the current construction industry [34].

The environmental repercussions of construction projects include a substantial contribution to waste generation, notably in the form of illegal dumping. The increasing incidence of illicit dumping associated with construction endeavors in Malaysia underscores the pressing need for comprehensive management of construction waste in the country [35]. However, it is noteworthy that there is currently a lack of a well-defined set of criteria for construction waste management, particularly within developing nations. The incorporation of 3R's methodologies, namely Reduction, Reuse, and Recycling, into the domain of materials management [32]. In addition, it examines the potential applicability of these 3R principles across various industries, including the construction sector [28]. Furthermore, materials management holds a paramount position in enhancing operational efficiency within construction projects.

A study by Ref. [36] stated that all 105 participants from “Georgetown, Penang, Malaysia”, took part in the survey. The hawker community's perception, knowledge, and practicing of solid waste disposal was examined using a descriptive approach; this study focused on composting and recycling. The conclusions of this study are intended to provide helpful information and relevant viewpoints for constructing operators, which may be altered to further enhance recycling processes. The majority of respondents (51.4%) found recycling operations to be unpleasant and displeasing.

The utilization of sustainable and rounded materials, which encompass locally sourced and recycled materials, offers the potential to reduce vulnerabilities in the supply chain while simultaneously fostering the principles of environmental sustainability. The incorporation of energy-efficient systems, designed to connect the unique attributes of the construction sector, can effectively reduce operational risks while advancing the material management objectives [37]. Additionally, it is of dominant importance to acknowledge the significance of identifying context-specific approaches, transferable knowledge, and best practices that may deviate from those observed in regions such as Hong Kong and New Zealand, and to thoughtfully apply these principles when extending them to diverse international contexts. The past studies regarding the 3R practice as material waste reduction are significant since the contractors identified it as an efficient and effective strategy to decrease material waste in a construction site [38].

Reused building materials are sourced from various stages of building construction, maintenance, and demolition activities, and they can be readily incorporated into other projects following minor refurbishment. On the other hand, the recycling approach for construction waste involves the disassembly of building components, which are then blended with other materials to create fresh construction materials. This practice effectively minimizes construction waste generation and conserves valuable construction resources. Both the reuse and recycling methods of managing construction waste contribute to a reduction in carbon emissions associated with construction processes and yield substantial economic and environmental advantages [39]. To preserve our planet's finite natural resources, we must take steps to reduce material waste from its origin as the 3R approach is regarded as a promising strategy to both prolong the operational lifespan of landfills and lessen the necessity for extensive natural resource extraction [5].

[32] Indicates that the (3R) practice outlines specific waste actions, while [29]defines it as obligatory for all involved parties during construction. Enhancing high-quality material production through 3R practices includes reducing on-site material cutting, a key strategy for waste reduction [40]. Another justification by Ref. [41] stated that applying such a modern method technology for construction waste material is considered a strategy that has to be applied by 3R during the construction of materials, while [42] in their study developed that 3R practice used to monitor the waste generation during the implementation of materials in construction project. Reusing construction materials can effectively reduce waste generation in construction processes while also improving site management due to waste reduction [43]. The recycling of construction waste holds the potential to efficiently reintegrate valuable resources into the production cycle, consequently mitigating waste treatment expenses before disposal [44]. Furthermore, this practice not only aligns with environmental conservation objectives but also contributes to reducing the strain on land resources designated for material waste disposal. Therefore, the construction industry should focus on the 3R strategy to ensure adherence and improve construction site performance.

#### 2.1.2 Material waste mitigation utilizing Industrial Building Systems (IBS)

The Industrialized Building System (IBS) has gained acknowledgment as a practical approach for enhancing overall construction performance concerning quality, cost-effectiveness, safety and health standards, waste reduction, and productivity. In Malaysia, the construction industry is regarded as a significant contributor to the nation's development [45]. According to the Construction Industry Development Board Malaysia (CIDB), IBS is described as the method of producing construction components within a controlled environment, then positioning, transporting, and assembling them into a structure with minimal additional on-site activities [46].

To enhance the performance of the construction sector, innovative approaches have been introduced to enhance its efficiency. Among the prominent methods being employed and researched are Industrialized Building Systems (IBS) and prefabrication construction techniques [47]. The utilization of (IBS) technology, which emphasizes off-site prefabrication and modularization, not only leads to a substantial decrease in environmental harm but also results in substantial productivity improvements, reduced labour demands, and enhanced working conditions. IBS technology has now become a widespread and expanding construction method in both developed and developing nations [48].

IBS has been identified as an effective approach for waste reduction on construction sites. A study conducted by Ref. [47] compared waste materials between IBS projects and conventional projects, revealing that in conventional projects, waste production was predominantly attributed to steel bars (50.25%), miscellaneous items (47.34%), and timber (2.21%). In contrast, IBS projects generated significantly lower waste percentages, with 0.77% for timber, 13.01% for steel bars, and 19.98% for miscellaneous waste [49].

An industrialized building system IBS is a construction method that makes use of tools, materials, or structural components to be installed as prefabricated parts at the worksite. IBS application in construction contributes to improving both quality and productivity [50]. Another research by Ref. [51] pointed out that IBS was used in construction to minimize wastage in a construction site, while [51] in their finding stated that IBS was used to minimize the use of timber form in the construction site. Furthermore [52], developed that IBS implementation is significant for reducing noise by providing ready elements to be installed in the working place. According to the benefits of IBS use for construction waste reduction and its economical usage that lowers the total construction costs stated by Ref. [53], industrialized building systems (IBS) practice is gaining increasing attention for a construction site, particularly as it reduces the dependency on foreign labors, and site labor numbers as well, the finding may hypothesis that IBS technology and its innovation in construction site might be one of the most efficient ways to improve the performance of a building site.

#### 2.1.3 Material waste mitigation utilizing Building Information Modeling BIM

BIM exhibits the capacity to function as an auxiliary technology for the mitigation of risk factors impacting waste generation. Furthermore, Building Information Modeling BIM facilitates collaborative efforts and streamlines design and construction processes by connecting cutting-edge 3D modelling software, providing open access to information, and promoting multidisciplinary integration within the context of waste management [54]. According to research by Ref. [55], it is asserted that Building Information Modelling (BIM), functioning as a digital technology platform, presents auspicious avenues for enhancing the widespread implementation of waste management strategies. The incorporation of BIM in such a manner stands to potentially mitigate uncertainties of waste prediction and management in construction projects. In the realm of digital technology platforms, Building Information Modeling (BIM) not only holds considerable promise for enhancing waste reduction and control but also for fostering the wider implementation of waste management techniques within the construction sector [56]. BIM enhances communication, fosters efficiency, and reduces errors, thereby contributing to resource conservation, energy efficiency, material preservation, and waste reduction. Additionally, BIM empowers users to promptly revise, assess, reject, or endorse design concepts in a real-time environment [57].

Proper material storage methods and locations, efficient site utilization planning, and strategic site layout planning all contribute to minimizing material waste by optimizing the quantity and distance of material handling activities, leveraging a 4D model, and implementing waste reduction strategies like recycling and reuse throughout the entire project duration. Managers can also utilize 3D-BIM to scrutinize site layout plans against predefined regulations, ensuring a conflict-free alignment with the design before commencing construction [57]. The incorporation of prefabricated components holds the potential to reduce waste by as much as 52%. This is due to the precision of measurements provided by BIM during the initial design phase, enabling fabricators to better regulate and automate the fabrication process [58]. The production of prefabricated elements can yield higher-quality outputs at reduced costs. Overall, this approach proves effective in averting delays stemming from the time spent searching for suitable components or encountering late material deliveries. By employing BIM, all aspects of a project can be consolidated into a unified model that encompasses comprehensive model-related information, including designs, costs, and materials utilized, among others. This data is seamlessly shared and kept current among all stakeholders, streamlining traditional communication practices. Consequently, it enhances the delivery of information in terms of its content, clarity, and speed [59].

BIM is used as one of the construction aspects because of its model-based qualities and the technology for coordinating the usage of construction knowledge, it is rising in favour, and that related to its indicators of construction firm advantageous properties provided more emphasis to reducing the costs and delivery time, while quality improvement and sustainable construction were also given significant effect [60]. In recent years, BIM has been widely regarded as a game-changing innovation in the building sector. The abbreviation term BIM is the acronym for “building information modelling,” which is a set of technologies and related works that are used to define and manage the information needed and created throughout the building design, construction, and operation processes [30].

[61] Highlighted that BIM technology plays a positive role in fostering communication and collaboration among project personnel, expediting project timelines, minimizing material waste, reducing costs, and enhancing project sustainability. It is evident from the work of past academics and experts in the field that BIM technology can boost project efficiency, reduce mistakes, and save both time and cost [62]. Overall productivity improvements are among the primary advantages of using BIM. High performance in construction is attainable because of BIM's adaptability to a variety of management procedures and studies, which include both the operational and financial aspects of a project [63]. Low productivity, little technical innovation, and computerization are described in the construction business as a study by Ref. [64] stated that Advanced technologies like BIM can boost efficiency, cut down on expenses, and speed up the completion of construction projects while similarly, research by Ref. [65] indicated that, BIM has been gaining some traction in Singapore's construction sector as a way to more effectively coordinate stakeholders in the process, boost site performance, and cut down on rework.

#### 2.1.4 Material waste mitigation utilizing Material Management Adoption (MMA)

Effective material management is a critical aspect of project productivity as material management strategies encompass systematic methods that revolve around the planning, organization, and control of materials in the construction site [43]. Material storage encompasses receiving initial materials at the construction site, organizing, and storing them for a designated duration until they are needed for work. This process is recurrent, especially in congested construction sites [66].

Planning involves determining the material requirements for production and related construction processes, including specifying the types, quantities, and specifications of materials needed. Material managers also have a crucial role in deciding storage locations, and layouts and acquiring all necessary equipment, such as coding and cataloging systems, material acceptance procedures, material inspection protocols, storage safety measures, and maintaining stock records [2]. Adequate storage facilities must be provided on-site for materials, and close monitoring is essential to track quantities and prevent theft. Furthermore, construction quality plays a pivotal role in determining project acceptance and contractual payment levels. Participants in the construction industry have become increasingly aware of the significance of quality in ensuring client satisfaction and gaining a competitive edge [67].

The wide range and quantity of available resources, the range of tasks that each required team is capable of performing, the efficiency of each required work associated with costs, and the distribution pattern of all resource management over different locations, resulting in a need for relocation from one site to another, all increase the complexity of managing resources in the worksites [68]. Supply-chain management, which is only focused on controlling materials in real-time at the construction sites for their purchase, storing, and delivery, is an automated technology that has been used in several aspects of building project management so far [69]. The strategy for obtaining the requisite performance in construction projects, known as site management, became more widely accepted. This is due to a realization that appropriate site management is necessary for achieving performance [70]. One of the most important criteria for improving waste reduction is to increase on-site material reuse [71]. A study by Ref. [72], Particularly where resource management and contact factors methods highlights the significance of cultivating a workforce engaged in environmental concerns, which may enhance performance effectively promoting green culture relies on cooperation, workforce training, assessment and evaluation of environmental objectives, and corporate culture and competitive performance to enhance construction site performance. Minimizing costly materials usually positively enhances construction site performance from quality assurance and economic perspectives [73].

Performance measures rely on having the appropriate people with the right skills and equipment to produce projects on schedule and within budget and having the right materials in the proper location [14]. Income stream and capital are also given careful attention to purchasing the materials and labour [74]. At the same time, site performance is enhanced positively by estimation, budgeting, planning, and monitoring materials in the site workplace [2,75].

Scheduling of materials through material management provides an overview of the time-related work required to finish the project [76]. They add that material management positively affects material handling and transport on the construction site. Besides, quality assurance is one material management that affects the construction site's cost, time, and quality performance. Another study by Refs. [77,78] developed that receiving and inspection are the material management indicators that enable store managers to organize and plan for clearances of materials on site [79]. Emphasized that inventory control, storage, and warehousing are crucial components contributing to construction site performance. By taking into account various degrees of construction material waste mitigation to enhance construction site performance, knowledge sharing, on the other hand, substantially moderates the association between technology adoption and site performance.

### 2.2 Research hypothesis

The construction business has always been at the forefront of technological advancements, and this trend has only accelerated in recent years, especially when one considers the context of the numerous new technologies that have been developed specifically for this sector [1]. A study by Ref. [2] states that (3R) practice normally specifies the action for each type of waste, while [5] in their finding defined 3R practice in the construction site as responsibilities that all parties involved in the site have to consider as important action to follow during construction workplace. Maximizing the production of high-quality materials takes action 3R practice and reducing the onsite cutting of materials is one of the 3R practice strategies toward waste mitigation measures [40]. Another justification by Ref. [4] stated that applying such a modern method technology for construction waste material is considered a strategy that has to be applied by 3R during the construction of materials, while [9] in their study developed that 3R practice used to monitor the waste generation during the implementation of materials in construction project. Reusing construction materials can effectively reduce waste generation in construction processes while also improving site management due to waste reduction [43]. Therefore, the construction industry should focus on the 3R strategy to ensure adherence and improve construction site performance.

H1 Is there a significant relationship between material management practices utilizing the (3R) approach as material waste mitigation and (CSP)?

Innovations in both technology and construction technology had a major impact on the construction sector. As a result of IBS's widespread implementation of IBS, Malaysia's construction sector witnessed a major transformation which has contributed to its success [8]. Other related performance of IBS as well as the actual construction processes and components in production. An industrialized building system IBS is a construction method that makes use of tools, materials, or structural components to be installed as prefabricated parts at the worksite. IBS application in construction contributes to improving both quality and productivity [11]. Another research by Ref. [12] pointed out that IBS was used in construction to minimal wastage in a construction site, while [24] in their finding stated that IBS was used to minimize from using timber form in the construction site. Furthermore [13], developed that IBS implementation is significant for reducing noise by providing ready elements to be installed in the workplace. Through careful design and execution, prefabricated elements might decrease the quantity of waste on the worksite. According to the benefits of IBS use for construction waste reduction and its economical usage that lowers the total construction costs stated, industrialized building systems (IBS) practice is gaining increasing attention for a construction site, particularly as it reduces the dependency on foreign labors, and site labor numbers as well, the finding may hypothesis that IBS technology and its innovation in construction site might possibly be one of the most efficient ways to improve the performance of a building site.

H2 Is there a significant relationship between Technology adoption utilizing (IBS) as material waste mitigation and (CSP)?

In recent years, BIM has been widely regarded as a game-changing innovation in the building sector. The abbreviation term BIM is the acronym for “building information modelling,” which is a set of technologies and related works that are used to define and manage the information needed and created throughout the building design, construction, and operation processes [80,81]. According to Ref. [17] BIM technology positively ensures that project personnel are communicating and working together to speed up the projects and contribute to maximizing the material waste while decreasing the costs and boosting the sustainability of the project. It is evident from the work of past academics and experts in the field that BIM technology can boost project efficiency, reduce mistakes, and save both time and cost [18]. Low productivity, little technical innovation, minimal automation, and computerization are described in the construction business as the study by Ref. [19] stated that Advanced technologies like BIM can boost efficiency, cut down on expenses, and speed up the completion of construction projects while similarly, research by Ref. [20] indicated that, (BIM) has been gaining some traction in Singapore's construction sector as a way to more effectively coordinate stakeholders in the process, boost site performance, and cut down on rework. The value of building information modelling (BIM) to the construction industry's development and efficiency has been widely acknowledged. In this way, BIM facilitates stakeholder collaboration, vision, and appropriate management of construction projects by planners, suppliers, and business managers.

H3 *Is there a significant relationship between Technology adoption utilizing (BIM) as material waste mitigation and (CSP)?*

Performance measures rely on having the appropriate people with the right skills and equipment to produce projects on schedule and within budget and having the right materials in the proper location. Income stream and capital are also given careful attention to purchase the materials and labor. At the same time, site performance is enhanced positively by estimation, budgeting, planning, and monitoring materials in the site workplace [49]. As [32] stated scheduling of materials through material management provides an overview of time-related work required to finish the project. They add that material management positively affects material handling and transport in the construction site. Besides, quality assurance is one material management that affects the construction site's cost, time, and quality performance. Another study by Ref. [33] developed that receiving and inspection is one of the material management indicators that enable store managers to organize and plan for clearances of materials on site [35]. in their study regarding construction site management, stated that inventory control, storage, and warehousing are essential for construction site performance. Furthermore, in contrast, the design, production, shipping, placing, and assembly of the constructions have such a sound effect that the extra site work is kept to a minimum [53]. Site communication is one material management indicator that provides sufficient information between all parties. It was shown to impact building site performance significantly [37]. Project teams with different values and interests. Effective collaboration and communication between all stakeholders are essential, given the nature of the work. Besides [39], in their finding they mentioned that value engineering is one of the material management aspects with higher specifications and less cost for construction site management. By considering various degrees of construction waste mitigation to enhance construction site performance, knowledge sharing, on the other hand, substantially moderates the association between automation technology adoption and site performance.

H4 Is there a significant relationship between (MMA) as material waste mitigation and (CSP)?

The Malaysian government has encouraged the principle of 3R by implementing various laws and procedures through waste management practices among developers and professionals to accept materials. Although government bodies have implemented certain regulations and procedures, it is insufficient in their practical image [34]. However, inadequate market development, on the other hand, demonstrates that substantial amounts of resources and management are required to form partnerships and monitor pricing fluctuations to become a competent materials supplier that provides a continuous flow of essential supplies. As a result, insufficient waste recycling markets will severely restrict the effective implementation of waste reusing as a comprehensive waste management solution. It also preserves landfill space and reduces the number of landfills and their related expenses, along with energy savings and environmental damage reductions. Construction management solutions are more likely to save resources and improve the environment, according to a proactive approach. Therefore, it can be inferred that the industry requires an automated instrument for management, particularly one that would be extremely friendly and convenient.

H5 Material *Management Adoption (MMA) moderates the relationship between (3R) and (CSP).*

Materials management in construction recently adopted various technologies in the firm; IBS technology is a modern advanced technology [25]. The Malaysian construction industry enjoys the advantages of producing higher structures, on-time construction finishing, and cost savings through the uniformity of IBS. On the other side, development is employed to design the overarching strategy for the construction industry [11]. As a result, the developer needs to work closely to address their efforts throughout the implementation stage to attain a better efficiency level. However, evaluating the amount of cooperation in construction companies has proven to be challenging.

Furthermore, performance must be measured systematically to assist the relevant parties in determining the contractor's competence and commitment to complete the duties given [82,83]. The evaluation will also assist in identifying areas that need improvement and measure how the participants feel about the finished image or outcome. In sustainable buildings, actual environmental performance is crucial, and the inadequate monitoring of ecological performance may impede workers' performance. Environmental obligations, issues, policies, green information systems, and employee assessment should be the common subjects of a green performance evaluation through implementing IBS and its benefits in such material and cost saving. These areas should cover job-related requirements and volunteer external and internal environmental initiatives [84,85].

According to Ref. [45] states that improper resource utilization impacts several factors, including but not limited to budgets, profits, project quality, timeliness, etc. Significant construction delays have severe consequences, including cost overruns and decreasing profitability margins. In conclusion, the existing industry's fragmentation is a significant issue for the continued use of conventional design and management for the resources, which required such innovation through the adoption of automation technology in construction firms toward the enhancement of overall projects.

H6 Material *Management Adoption (MMA) moderates the relationship between the (IBS) and (CSP)*.

The efficient implementation of construction works employing the BIM era of the twenty-first century should incorporate intelligent solutions to assist construction firm teams in making commitments to complete projects within major construction criteria, including cost, schedule, quality, and, more importantly, sustainability standards which commonly addressed by project team members [51]. Therefore, there is an urgent need for projects to incorporate BIM for managing material resources, especially in a large-scale construction site concerning many opportunities to plan and model improved material use and circular economy in the built environment. This themed issue aims to tackle these gaps in knowledge and it aims to promote and support the adoption of BIM technology and its practices to mitigate material waste in construction sites.

H7 *Material Management Adoption (MMA) moderates the relationship between (BIM) and (CSP).*

**Fig. 1:**
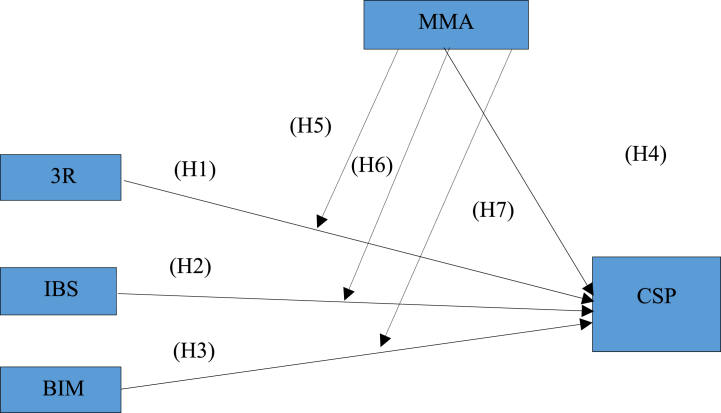
Conceptual framework.

To establish a foundational structure for our investigation, we have constructed a conceptual framework (illustrated in Fig. 1) rooted in prior studies and a comprehensive review of existing literature.

## 3 Research methodology

### 3.1 Philosophical stances

**Fig. 2:**
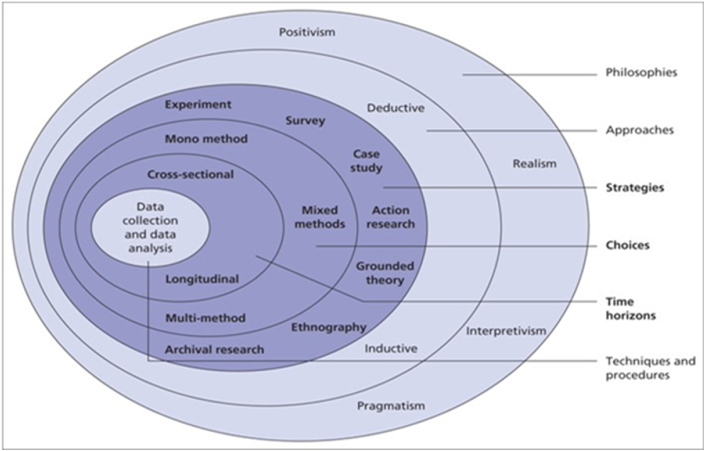
Methodology Research Onion Source [36].

The term “research methodology” encapsulates the overall approach used in conducting research, including underlying beliefs and philosophical assumptions shaping research questions and methods. It's a fundamental aspect of dissertations or theses, ensuring alignment between selected tools, techniques, and the underlying philosophy. One widely used model for structuring a research methodology is the “research onion,” proposed by Ref. [35]. This model delineates key stages in crafting an effective methodology, starting with defining the philosophy, selecting approaches and methods, determining time frames, and finally, forming the research design. In the social sciences, researchers employ various strategies, practices, and procedures. The research onion, as outlined by Refs. [35,36], visually represents these stages and strategies, illustrated in Fig. 2, utilized in this study to determine the research design.

The present study adopts a positivist approach, primarily due to its ability to utilize large sample sizes with minimal involvement of the researcher in the problem domain, thereby reducing any potential biases in the results [36]. Furthermore, this approach aligns well with the research objectives, which entail the development of hypotheses, their subsequent testing, and the generalization of results to the population. Additionally, the study follows a deductive approach, as it builds upon prior theories to establish the research hypotheses.

The research methodology employed in this study, rooted in the positivism paradigm, seeks an objective exploration of factors influencing material waste reduction in the construction industry [86]. The adoption of positivism aligns intending to uncover unbiased knowledge through empirical methods, emphasizing the importance of reliable and generalizable data. However, it's crucial to acknowledge the limitations inherent in this approach, as it might overlook subjective aspects of reality, a criticism often directed towards positivism. The study defends its choice by emphasizing the need for objectivity and the ability to generate valid and reliable data unaffected by human values. The progression from positivism to post-positivism and critical realism reflects a nuanced understanding of research philosophies [87,88], and [89]. Post-positivism's emphasis on understanding the causes behind actions rather than striving for absolute truth, and critical realism's acceptance of both objective and context-based realities, showcases a sophisticated approach to dealing with inherent biases in research [90].

Moving to research approaches, the study adopts a deductive stance, initiating from established theories to form hypotheses tested against empirical data. This choice, justified by the study's objectives and the desire for generalizability, aligns the research with a theory-driven hypothesis perspective. The selection of research strategy as prescriptive and descriptive, coupled with a quantitative approach using survey methods, aims to present objective outcomes. The emphasis is on employing the 3R approach, BIM, IBS technology, and Material Management Adoption (MMA) to assess Construction Site Performance (CSP) within the Malaysian context.

### 3.2 Research approach

In the methodological field, two main approaches to research exist. These approaches are positivist and interpretivist [91]. While the first one has been widely recognized as a scientific and quantitative approach, the second one has been considered a qualitative one. Although the main concern of the methodological approaches is almost identical, both have their own positive and negative effects on the research context by hook or crook [92]. According to Ref. [85], the positivist philosophy involves formulating and testing hypotheses, suitable for larger sample sizes, and explaining and predicting effects between variables. This research examines waste material mitigation's impact on construction site performance, moderated by MMA, aligning with this philosophy due to hypothesis testing on a sizable sample. It employs a quantitative approach to describe current conditions, effects, and relationships. Additionally, it aims to address gaps by comprehensively exploring technology and management approaches encompassing 3R, IBS, BIM, and Material Management Adaption (MMA) to mitigate material waste and improve construction site performance.

The research methodology adopted in this study aligns with a positivist approach, aiming to impartially uncover causal relationships affecting the phenomenon under investigation, as detailed in Table 1 [93]. The choice of this philosophy is rooted in its methodological values, such as rationality, objectivity, and control, which are vital for maintaining objectivity in data analysis and collection [94]. Positivism relies on established hypotheses, quantitative data collection, and objective analysis to understand specific phenomena and generalize findings [95].

**Table 1:** Summary of the research design source: Researcher (2023).

The Philosophy of the research	Positivism
Research Approach	Quantitative research
Time Horizons	Cross-Sectional
Research Strategy	Survey
Research Technique	Questionnaire
Research Analysis	Multivariate Analyses

This study utilized an online survey method to gather data due to practical constraints in reaching respondents at their workplaces. The researcher targeted construction practitioners in Malaysia using email lists derived from statistics provided by the Construction Industry Development Board (CIDB) for 2021. This industry consists of professionals like project managers, architects, contractors, engineers, and quantity surveyors. The study's sample size was determined based on guidelines referencing CIDB's records, indicating 769,351 registered construction personnel in Malaysia, including 131,121 contractors. Following established guidelines, the required sample size for this finite population amounted to 384 individuals [96,82].

The inadequate mitigation of material waste, resulting from limited technological utilization and management practices on Malaysian construction sites, significantly impacts construction site performance through causal and consequential factors. This study incorporates research indicators, including the initial adoption of the 3R principle. The inclusion of these elements draws upon prior studies by Refs. [8,28,33,38,40,83,84]. The subsequent measurements related to the adoption of IBS for material waste mitigation, indicating improved construction site performance in this study, were derived from prior research conducted by Refs. [85,86]. Additionally [53], demonstrated the significance of IBS implementation in reducing noise by providing prefabricated elements for installation in the workplace.

The third measurement of BIM utilization was also adopted from previous studies conducted by [ 98, 99]. Research by Ref. [55], asserts that Building Information Modeling (BIM), functioning as a digital technology platform, offers promising opportunities for enhancing the widespread implementation of material waste management. Incorporating BIM in this manner has the potential to mitigate uncertainties in waste prediction and management in construction projects [57].

The fourth measurement in this survey, focusing on the adoption of MMA in construction site operations, plays a pivotal role in project success. Highlighting effective scheduling of materials, material management offers valuable insights into project timelines and significantly enhances on-site material handling and transport [76]. Material management elements profoundly impact construction site performance. Efficient inventory control, storage, and logistics [97,98], alongside streamlined design, production, and assembly processes [53], reduce additional site work. Effective collaboration among diverse project teams is pivotal [99]. Value engineering, highlighted by Refs. [51,100], offers cost-effective management strategies. Smooth site operations rely on effective storage, transport management [101], and optimized inventory control [74]. Material management involves selecting, coordinating, and controlling materials for quality, timely delivery, and cost minimization [26,102]. MMA plays a significant moderating role in the relationship between technology, management approaches, and site performance concerning material waste mitigation efforts. The construct of CSP has been adapted based on insights from previous studies conducted by Refs. [43,54,66,71,76]. The evaluation was a five-point scale, 1 = Strongly Disagree, 2 = Disagree, 3 = Neither Agree nor Disagree, 4 = Agree, 5 = Strongly Agree [21]. After the researcher distributed the questionnaire and gathered the required amount of data, these questionnaires were keyed into the SPSS software. Many activities take place in this part, such as data entry, the screening of the collected data as well and the selection of the appropriate tests [103].

### 3.3 Research design, data collection, and analysis

Before the distribution of the questionnaire to construction personnel, expert validation was sought to refine, condense, or enhance its content. Industry experts, with over a decade of expertise encompassing academia, consultancy, contracting, and engineering within the Malaysian construction sector, offered invaluable insights. Their extensive industry knowledge significantly influenced the adaptation of questionnaire items Table 2. The questionnaire initially outlined instructions, research objectives, and demographic queries. Subsequently, it detailed factors impacting material waste mitigation through technological and management aspects within Malaysia's construction landscape, integrating expert-adapted items from previous studies. Comprising 54 adapted items measured on a five-point Likert scale, the questionnaire aimed to capture nuanced responses.The targeted respondents within the Malaysian construction industry are categorized as project managers, architects, contractors, engineers, and quantity surveyors, aligning with the latest CIDB classification update for the industry.

**Table 2:** Background of the professional experts in the Pre-Test.

No.	Expert	Affiliation	Recommendations
1.	Expert A	Academic	Shorten statements, remove similar/repeated questions
2.	Expert B	Contractor	Enhance demographics Ensure consistency in wording
3.	Expert C	Consultant	Increase font size for clarity Rewrite items for clarity Reduce questionnaires, simplify sentences
4.	Expert D	Academic	Edit English grammar Revise second section instructions
5.	Expert E	Academic	Include study introduction Clarify concepts Remove redundant item
6.	Expert F	Academic	Shorten survey length/pages and increase font size Improve online interface/layout
7.	Expert G	Engineer	Modify title format, reorganize Shorten survey

## 4 Data collection and results analysis

### 4.1 Respondents profile

The respondents, drawn from registered construction personnel within Malaysia's construction sector, offer a comprehensive representation of diverse stakeholders. Despite the study analyzing 295 respondents, accounting for 76.8% of the initially targeted sample size of 384, it remains an acceptable and inclusive sample from Malaysia's construction industry. Table 3 below provides a comprehensive demographic breakdown of participants involved in the Malaysian construction industry survey. It highlights their positions in the firm, organizational affiliations, project types, project costs, educational backgrounds, and experience levels.

**Table 3:** Demographic output, frequency, and percentage.

Category	Items	Frequency	Percentage (%)
Positions in the firm	Director	16	5.4
	Engineer	68	23.1
	Architect	85	28.8
	Project Manager	84	28.5
	Quantity Surveyor	35	11.9
	Academician/Researcher	5	1.7
	Other category	2	0.7
Organizational category	Designer/Consultant Firm	51	17.29
	Contractor	51	17.29
	Manufacturer	75	25.42
	Client	5	1.69
	Developer	54	18.31
	Local Authority/Government Agency	50	16.95
	Research/Academic Institution	16	5.42
	Other category	–	–
Project type	Building	85	28.8
	Infrastructure	60	20.3
	Institutional and Commercial	72	24.4
	Industrial	78	26.4
	Other category	–	–
Cost of project	<1 million Ringgit Malaysian (RM)	24	8.1
	1-5 million RM	29	9.8
	6-10 million RM	95	32.2
	11-15 million RM	87	29.5
	16-20 million RM	29	9.8
	>20 million RM	31	10.5
Highest level of education	Degree	56	19.0
	Diploma	46	15.6
	Master	102	34.6
	PhD	91	30.8
Years of experience	0–5 Years	15	5.1
	6–10 Years	70	23.7
	11–15 Years	78	26.4
	16–20 Years	103	34.9
	>20 years	29	9.8

The delineation of key roles in the Malaysian construction industry, as depicted in Table 3, showcases notable presences: Architects (28.8%), Project Managers (28.5%), Engineers (23.1%), and Contractors (25.4%). This distribution emphasizes their pivotal influence in project execution, material waste mitigation, and material management practices. Meanwhile, the organizational categories with substantial representation include Client (18.3%) and Developer (16.9%). In terms of project types, Building projects (28.8%) hold the highest percentage, followed closely by Industrial projects (26.4%). Regarding the cost of projects, significant proportions fall within the 6–10 million RM (32.2%) and 11–15 million RM (29.5%) ranges. Educationally, Master's degrees (34.6%) and PhD qualifications (30.8%) account for the highest percentages, while professionals with 16–20 years of experience (34.9%) constitute the most substantial expertise in the field.

### 4.2 Reliability test

Assessing the survey's variables for higher reliability signifies increased consistency across questionnaire sections and the overall mean. Cronbach's alpha coefficient ranges from 0.0 to 1.0, with higher values indicating better consistency. Table 4 presents the Cronbach's Alpha values for all variables, obtaining a score of 0.850, affirming strong reliability and validity across all items [104].

**Table 4:** Reliability test (Combined all variables).

Reliability Statistics	
Cronbach's Alpha	No. of Items
0.850	54

The reliability analysis in Table 5 highlights the consistency of key variables within the Malaysian construction industry among construction personnel. Notably, 3R, IBS, BIM, MMA, and CSP show strong internal consistency with high Cronbach's Alpha values (0.910, 0.898, 0.918, 0.930, and 0.933). These metrics are pivotal for assessing material waste reduction strategies, refining construction processes, and improving site performance in Malaysia's construction sector. These constructs are considered robust and consistent based on the established reliability criteria outlined in previous research theory [105,106].

**Table 5:** Test of reliability statistics (individual items).

Name of Variable	Cronbach's Alpha	No of Items
Construction Site Performance	0.933	15
Reduce, Reuse, and Recycle	0.910	9
Building Information Modeling	0.918	12
Material Management Adoptions	0.930	10
Industrial Building System	0.898	8

### 4.3 Mean and Std. deviation

The questionnaire results present descriptive statistics for the latent variables, showcasing the summary based on a 5-point Likert scale. This scale ranges from 1, indicating strong disagreement, to 5, signifying strong agreement [38]. Table 6, presents the results of the descriptive statistics below. The mean values of the factors exhibited a high significance level. The correlations among the factors were high, indicating strong differential validity and a high level of consistency between the factors' content tested and the overall questionnaire content [107].

**Table 6:** Descriptive statistics - means, standard deviations, skewness, and kurtosis.

Constructs	Mean	Std. Deviation	Skewness	Kurtosis
3R	3.76	0.50	−0.12	0.27
BIM	3.81	0.50	−0.25	0.31
IBS	3.82	0.53	−0.36	0.32
CSP	3.78	0.49	−0.53	1.88
MMA	3.74	0.57	−0.99	2.79

The mean scores for key construction factors 3R (3.76), BIM (3.81), IBS (3.82), CSP (3.78), and MMA (3.74) indicate a favorable perception of their significance. CSP shows a more concentrated response pattern with higher kurtosis (1.88), while MMA displays relatively higher kurtosis (2.79). Overall, these findings suggest a moderately positive view of these factors' importance in optimizing material usage and enhancing construction site performance within this context most researchers agree that a data set is considered normal if both skewness and kurtosis fall within the range of −3.0 to +3.0. Therefore, these values serve as the suggested threshold, denoting the acceptable limits for normality. The findings indicate a non-normal distribution of data, as all significance values are below 0.05, as shown in Table 7 below. Opting for PLS-SEM over covariance-based SEM was due to the distribution's non-normality, as PLS-SEM is more resilient to such distributions.

**Table 7:** Normal distribution test.

Constructs	Kolmogorov-Smirnov	Shapiro-Wilk
3R	0.116	0.771
BIM	0.245	0.867
IBS	0.251	0.867
MMA	0.220	0.871

The evaluation of multivariate collinearity relies on the Variance Inflation Factor (VIF), where a value exceeding 4.0 indicates collinearity concerns [104]. In Table 8, all VIF values below a certain threshold confirm the absence of multicollinearity issues in the data analysis results.

**Table 8:** Statistics related to collinearity: Variance Inflation Factors (VIF).

Construct	3R	BIM	IBS	MMA
VIF	1.167	1.567	2.337	2.465

### 4.4 The assessment of the PLS-SEM model

This study followed a two-stage method and presented PLS-SEM results [39], aligning with advice from Ref. [40] supporting the use of the goodness-of-fit (GoF) index over alternative nonparametric methods like bootstrap and blindfolding [41]. This approach provides a thorough evaluation of the PLS-SEM pathway model, offering deeper insights into the relationship between material waste mitigation and construction site performance.

#### 4.4.1 Assessment of the model measurement

**Fig. 3:**
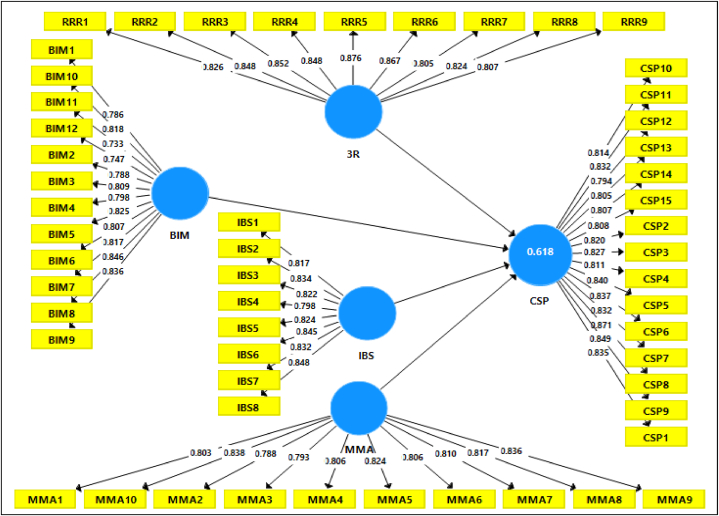
Mesurement model.

PLS-SEM evaluates the measurement model (outer model), gauging its relevance through three key aspects: (1) assessing individual item reliability using composite reliability (CR) for internal and indicator reliability, (2) evaluating the research instrument's convergent validity with the average variance obtained (AVE) related to independent factors, and (3) employing Fornell-Larcker criteria and indicator outer load conditions to ensure discriminant validity [41,42]. This thorough examination confirms the precision and trustworthiness of the measurements. The conclusive and accurate measurement model, created using Smart-PLS is illustrated in Fig. 3.

#### 4.4.2 The individual items reliability

According to Ref. [41] the convergent validity is attained when the factor loading across all items is greater than 0.50. The rule of thumb for deciding whether to keep or remove an item is to keep the items with loading values of 0.50 and 0.70 [41]. As a result, the factor loading of all constructs 54 model elements varied from 0.733 to 0.876. The results are presented in Table 9 below.

**Table 9:** Reliability reflective, loading, average variance extracted of constructs summary.

Constructs	Item	Loadings >0.70	Cronbach's Alpha	Composite Reliability	AVE >0.5
Construction Site Performance	CSP1	0.835	0.947	0.970	0.682
	CSP2	0.820			
	CSP3	0.827			
	CSP4	0.811			
	CSP5	0.840			
	CSP6	0.837			
	CSP7	0.832			
	CSP8	0.871			
	CSP9	0.849			
	CSP10	0.814			
	CSP11	0.832			
	CSP12	0.794			
	CSP13	0.805			
	CSP14	0.807			
	CSP15	0.808			
Reduce, Reuse, and Recycle	3R1	0.826	0.948	0.956	0.705
	3R2	0.848			
	3R3	0.852			
	3R4	0.848			
	3R5	0.876			
	3R6	0.867			
	3R7	0.805			
	3R8	0.824			
	3R9	0.807			
Building Information Modeling	BIM1	0.786	0.949	0.956	0.643
	BIM2	0.788			
	BIM3	0.809			
	BIM4	0.798			
	BIM5	0.825			
	BIM6	0.807			
	BIM7	0.817			
	BIM8	0.846			
	BIM9	0.836			
	BIM10	0.818			
	BIM11	0.733			
	BIM12	0.747			
Industrial Building System	IBS1	0.817	0.934	0.946	0.685
	IBS2	0.834			
	IBS3	0.822			
	IBS4	0.798			
	IBS5	0.824			
	IBS6	0.845			
	IBS7	0.832			
	IBS8	0.848			
Material Management	MMA1	0.803	0.943	0.951	0.660
	MMA2	0.788			
	MMA3	0.793			
	MMA4	0.806			
	MMA5	0.824			
	MMA6	0.806			
	MMA7	0.81			
	MMA8	0.817			
	MMA9	0.836			
	MMA10	0.838			

The results demonstrate that all variables exhibit a Cronbach's alpha exceeding 0.70, indicating consistency within the investigated variables [43]. Moreover, the table illustrates robust reliability among all factors, as reflected by their AVEs surpassing the threshold value of >0.5, affirming the reliability of the measurement model [45]. The level of acceptance is contingent upon the Kaiser-Meyer-Olkin (KMO) values, typically deemed mediocre between 0.5 and 0.7, good between 0.7 and 0.8, great between 0.8 and 0.9, and superb above 0.9 [108]. In this study, both KMO and Bartlett's test, along with factor analysis, were executed using SPSS. The value obtained for all constructions was 0.894, surpassing the recommended threshold of 0.6 [105]. In conclusion, the questionnaire developed in this study demonstrates good reliability and validity.

#### 4.4.3 Internal consistency reliability

The internal consistency of reliability measures the extent to which all elements gauge various aspects or scales [46]. Cronbach's Alpha (CA) and Composite Reliability (CR) coefficients are commonly utilized in material management research to assess the internal validity and consistency of scales, especially those with multiple items [108]. In this study, the latent variables exhibited satisfactory internal consistency, with Cronbach's Alpha (CA) values ranging from 0.934 to 0.949 and Composite Reliability (CR) values from 0.946 to 0.970. These values surpass the minimum threshold of 0.70 in Table 9 [46].

#### 4.4.4 Convergent validity

Convergent validity (CV) assesses the degree to which items align with intended latent constructs and their association with other measures of similar constructs [105]. As per [48] achieving a satisfactory CV necessitates an AVE of 0.50 or above for each variable, while composite reliability should reach 0.70. Table 9 demonstrates AVE values ranging between 0.643 and 0.705. Hence, it is evident that each construct in this study establishes convergent validity, given that each item sufficiently mirrors the latent components.

#### 4.4.5 Discriminant validity

Table 10 illustrates the correlations between latent variables and their corresponding Average Variance Extracted (AVE) square roots. Following the guidelines outlined in Ref. [50], it is observed that the diagonal values, representing each item's AVE, exceed the values within their respective columns and rows, thus affirming discriminant validity.

**Table 10:** Root square of AVE (Fornel and Larcker result).

Constructs	3R	BIM	IBS	CSP	MMA
3R	**0.840**				
BIM	0.195	**0.802**			
IBS	0.557	0.49	**0.826**		
CSP	0.438	0.384	0.615	**0.828**	
MMA	0.427	0.292	0.584	0.363	**0.812**

The HTMT values in Table 11 are below 0.85, confirming discriminant validity without multicollinearity concerns among latent constructs, indicating no overlapping elements were used to measure the same construct. As a general rule, discriminative validity occurs if the value is higher than the restrictive value of 0.85 [105].

**Table 11:** Heterotrait-Monotrait Ratio (HTMT).

	3R	BIM	IBS	CSP	MMA
3R					
BIM	0.200				
IBS	0.577	0.510			
CSP	0.46	0.403	0.641		
MMA	0.449	0.308	0.609	0.382	

### 4.5 Structural model the direct relationships

The structure model consists of; (i). Evaluating the testing of hypotheses, (ii) Evaluation of the Variance Explained by Endogenous Latent Constructs, (iii) Evaluation Coefficient of Determination R^2^ with the effect size (f^2^), and (iv) Evaluation of the model fit by Predictive Relevance (Q^2^) [[108], [109], [110]].

#### 4.5.1 Assessment of hypothesis testing (path coefficient)

**Fig. 4:**
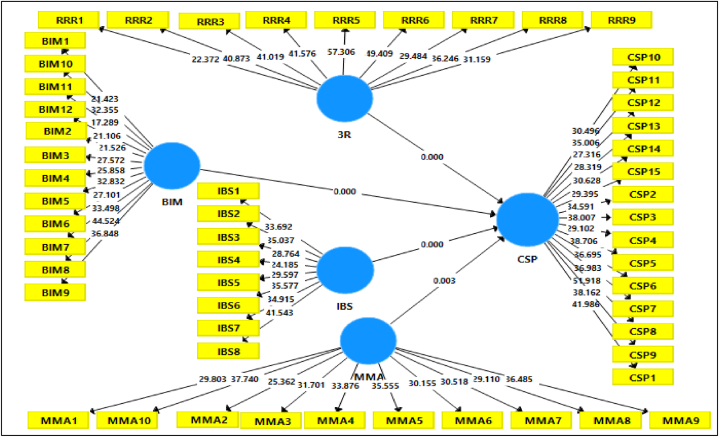
Structural model direct relationship.

Table 12 and Fig. 4 present the results of direct relationships between technology and management adoptions towards Construction Site Performance (CSP), indicating significant support for each relationship.

**Table 12:** Result of direct relationship.

No	Relationship	Std. Beta	Std.Dev	T- values	P-values	Decision
H1	3R - > CSP	0.250	0.067	3.753	0.000	Supported **
H2	BIM - > CSP	0.237	0.065	3.652	0.000	Supported **
H3	IBS - > CSP	0.307	0.083	3.700	0.000	Supported **
H4	MMA - > CSP	0.296	0.101	2.942	0.003	Supported *

Significant at P*<0.05; P**<0.01.

#### 4.5.2 Coefficient of determination (R^2^)

The criteria for the R^2^ level are proposed by Ref. [111]. If the values of R^2^ are more than 0.67, the result indicates a high level, while if the values are between 0.33 and 0.67, the justification indicates a moderate level. In addition, if the value of R^2^ obtained a range of 0.19–0.33, then the level is considered as a small portion, and if the values of R^2^ indicate 0.19 and less, then the evaluation is deemed to be unacceptable. As a consequence of the findings of this research, the R^2^ values have matched [111,112,113] requirements at a moderate level of 61.8. The R^2^ values are presented in Table 13 below.

**Table 13:** Variance explained in the endogenous construct.

Latent construct	Variance explained (R^2^)
Construction Site Performance	0.618%

#### 4.5.3 Assessment of effect size (f^2^)

The f^2^ value of the endogenous latent component is used to assess the strength of a model. Furthermore, this approach is appropriate for assessing the magnitude of the influence or contributions of the exogenous construct out of the proposed endogenous variable to determine the extent to which the exogenous constructs contribute to the exogenous constructs. By “running a PLS algorithm” with one exogenous component removed from the model, researchers can obtain the f^2^ excluded value for the same variable. To obtain the f^2^ included value, the procedure is repeated with the other variable in the model [52,53]. Effect size (f^2^) is determined by formula (1) utilizing the observed changes in R^2^ [53,107].

(1) $$f^{2}=\frac{R_{in}^{2}-R_{ex\;}^{2}}{1-R_{ex\;}^{2}}$$

As a result, values more than 0.35 are rated as significantly high, and if the values between 0.15 and 0.35 have to be considered medium, while the values between 0.02 and 0.15 are considered small, while if values less than 0.02 are reasoned to have no impact as guided by [ [52,53]].

According to Table 14, the f^2^ of 3R and effects on CSP were small whereas, the f^2^ of BIM and MMA on CSP was medium as guided by [ [[42], [43], [45], [46], [47], [48], [50], [51], [52], [53], [111]]]. Furthermore, this illustrates the significant impact of Technology and management adoption as material waste mitigation in Malaysian construction sites.

**Table 14:** Effect size (f^2^) for the latent exogenous construct.

Latent Construct	R^2^-Included	R^2^- Excluded	F^2^	Decision
3R	0.618	0.573	0.119	Small
BIM	0.618	0.573	0.121	Small
IBS	0.618	0.552	0.172	Medium
MMA	0.618	0.552	0.174	Medium

#### 4.5.4 Predicative relevance (Q^2^)

The blindfolding technique was employed to compute Q^2^, determining cross-validity redundancy or commonality. As previously mentioned, it aligns with the PLS-SEM approach. A positive cross-validity redundancy value denotes predictive relevance, while a negative Q^2^ value indicates a lack of productiveness [ [[42], [43], [45]]]. The Q^2^ results are detailed in Table 15 below.

**Table 15:** Predicative relevance Q.^2^.

Q^2^	SSO	SSE	1-SSE/SSO
Construction Site Performance	4425	2599.557	0.413

The results in Table 15 display the “sum of squared observations” (SSO), commonly known as SSO, and the “sum of squared prediction errors” (SSE), commonly known as SSE. Consequently, the endogenous latent variable (CSP) in this model has a Q^2^ value of 0.413, indicating an integer value greater than zero. This suggests that the model possesses “predictive relevance,” as previously emphasized by [45].

### 4.6 Testing the moderating effect

**Fig. 5:**
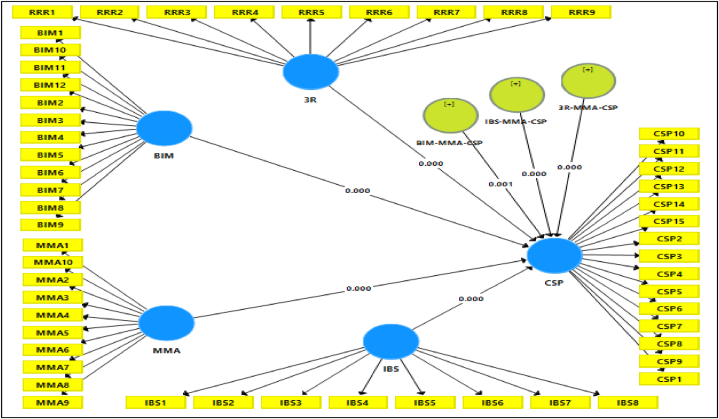
Moderation interaction effects.

The moderator variable and exogenous latent variables are both assessed reflectively in this research because the product indicator mechanism is applicable [53]. The specific indirect effect (moderator) findings are presented in Table 16 and Fig. 5 below.

**Table 16:** Results of the specific indirect effects.

NO	Relationship	Std. Beta	Std. Error	T- value	P-value	Decision
H5	3R- > MMA- > CSP	−0.240	0.054	4.429	0.000	Supported
H6	BIM - > MMA - > CSP	0.121	0.036	3.395	0.001	Supported
H7	IBS- > MMA - > CSP	0.213	0.052	4.118	0.000	Supported

Significant at P*<0.05; P**<0.01.

## 5 Discussion

The incorporation of diverse survey methodologies within this research holds promise for extending the reach of results within the construction sector. This presents an opportunity for future researchers to enhance data quality by employing similar survey techniques. Thoroughly examining measurements from varied contexts in the literature review underscores the need for a comprehensive validation of the scales' reliability and validity. Additionally, exploring construction personnel's understanding of material management and technology in the Malaysian construction industry addresses an under-explored aspect within the field.

Methodologically, this study aligns itself with existing knowledge by considering philosophical stances and research approaches. It particularly emphasizes the pivotal role of the construction site in exploring the impact of technology and material management on site enhancement. The methodology section elucidates the adoption of research factors, validating their contribution to existing knowledge on material management and construction site performance.

The research design involves a robust sample of 295 respondents from various roles within the Malaysian construction sector, representing 76.8% of the initial target sample size. Employing a two-stage–multi-analytic approach using SEM–PLS, the study identifies and validates factors influencing material waste mitigation in the Malaysian construction industry. It accentuates technological and managerial approaches aimed at enhancing industry performance and productivity.

The findings presented in Table 12 and Fig. 4 of the study highlight a strong positive association between material waste mitigation, particularly the 3R approach, and Construction Site Performance (CSP). Supported by empirical values (β = 0.250, t = 3.753, p < 0.000), this underscores the efficacy of 3R strategies in reducing construction material waste within the Malaysian industry. Similarly, the study confirms the influential role of BIM and Industrial Building Systems (IBS) in enhancing CSP (β = 0.237, t = 3.652, p < 0.000 for BIM; β = 0.307, t = = 3.700, p < 0.000 for IBS). Additionally, the direct association of MMA (Material Management Adoption) demonstrated significance (β = 0.296, t = 2.942, p < 0.003).

Certainly, the study emphasizes the significance of waste mitigation in Malaysian construction sites, particularly through the adoption of the 3R approach, BIM, IBS, and efficient material management practices. The ‘3R’ approach, focuses on waste reduction, reuse, and recycling, and the finding is supported by Refs. [28,32,40,43,82,83] showcasing its effectiveness in minimizing material waste. While BIM emerges as a crucial tool for waste management reduction, supported by Refs. [114,115] BIM tools and aided in the construction site help to speed up the projects and contribute to maximizing the material waste while decreasing the costs and boosting the sustainability of the project. These approaches and strategies not only aid waste reduction but also enhance communication, efficiency, and error reduction, contributing significantly to resource conservation and material preservation in construction projects [116,117], and [118]. In addition, IBS plays a vital role in mitigating material waste and improving construction site efficiency, as highlighted by Refs. [85,86] that IBS as ready-to-install components significantly reduce wastage and noise while streamlining the construction process. Furthermore, effective material management, encompassing aspects like scheduling, quality assurance, and inventory control, emerges as crucial for material waste management at the Malaysian construction site as demonstrated by Refs. [68,75,79,93]. Furthermore, the study reveals the crucial role of Material Management Adoption (MMA) in moderating the relationships between material waste mitigation and CSP. The significance of this moderation is supported by Refs. [[76], [77], [79]] emphasizing how effective material management positively influences project planning, sourcing, and cost-effectiveness. Statistically significant relationships between these factors and CSP are supported by their standardized betas, t-values, and p-values, each exhibiting varying effect sizes (f^2^), ranging from small to medium, as shown in Table 14. Moreover, the coefficient of determination (R^2^) highlights that a substantial proportion (61.8%) of the variance in CSP can be explained by these factors, as indicated in Table 14 under the term “R^2^-Included".

The interrelation among these constructs is further elucidated through Table 11 (Heterotrait - Monotrait Ratio - HTMT) and Table 10 (Correlation of Variables - Root Square of AVE), both indicating acceptable discriminant validity. Additionally, Table 9 confirms strong reliability across all factors, surpassing the threshold values (>0.5) for Average Variance Extracted (AVE), reinforcing the robustness of the measurement model [119,106]. The findings from additional statistical tests in Table 7, Table 8, including the Kolmogorov-Smirnov and Shapiro-Wilk tests, confirm the normal distribution of constructs, thereby ensuring the reliability of the data [104]. In conclusion, the integration of the 3R approach, BIM, IBS, and efficient material management practices plays a pivotal role in mitigating material waste in Malaysian construction sites, offering effective waste reduction, enhanced construction efficiency, resource utilization, and overall project success. This research substantially contributes to existing knowledge by affirming the positive impact of MMA on construction site performance through various material waste mitigation strategies, fostering a deeper understanding of optimizing construction outcomes and promoting sustainability.

## 6 Conclusion

In summary, this study has pinpointed key factors that significantly influence construction site performance through material waste mitigation in the Malaysian construction sector, highlighting the pivotal roles of 3R, IBS, BIM, and MMA in enhancing CSP. Construction personnel must prioritize these factors in their material management strategies, especially considering the expert validation of these constructs in this study. The proposed strategies recommend stricter regulations governing CSP-related technologies and management practices, aiming to refine worksite plans and augment construction productivity.

Nevertheless, this study faced limitations that warrant consideration. It focused solely on implementing technology and management approaches in the Malaysian construction sector. However, the findings could serve as a benchmark for other developing countries in Southeast Asia experiencing similar construction industry expansions. Moreover, the study's predictive power, as indicated by the R^2^ value (61.8%), was constrained, suggesting that only a portion of the identified factors can be elucidated. Future research endeavors should seek additional factors to augment predictive capability and further refine recommendations for bolstering CSP in the Malaysian construction industry. The scope of this study is confined to the context of construction sites within Malaysia, specifically targeting the construction personnel's from the solid waste management sector. It emphasizes the augmentation of construction site performance through the implementation of adaptive strategies and methodologies aimed at mitigating material waste within Malaysian construction sites.

The practical implications of this study extend to construction technology and innovation corporations, providing valuable insights to elevate their operational practices. Furthermore, it holds promise for nurturing growth and development within the Malaysian construction sector. To conclude, this comprehensive research, encompassing an extensive literature review and survey, has presented credible responses to the research questions and findings. The integration of material waste mitigation and CSP is pivotal for enhancing construction site performance and facilitating sustainable construction practices. This study paves the way for promising future research avenues, delving deeper into the intricate dynamics among these factors in the Malaysian construction industry.

## 7 Study implication and contribution

The study's implications encompass both theory and practice. The findings deepen our comprehension of how material waste mitigation influences CSP in Malaysian construction. In practical terms, the identified factors emphasize a heightened focus on material management and CSP within 3R, IBS, BIM, and MMA, thereby bolstering material waste mitigation and enhancing construction productivity. In the Malaysian construction sector, this study holds the promise of economic and environmental benefits by advocating for material waste mitigation at construction sites. It serves as a practical guide for stakeholders and policymakers, emphasizing the advantages of adopting technology and management practices to mitigate material waste and improve CSP. The study's contributions are evident in identifying crucial factors and employing innovative methodologies like PLS-SEM to provide a comprehensive view of CSP elements.

## 8 Future direction

Future research could explore expanded material waste mitigation techniques beyond 3R, IBS, BIM, and MMA, providing a more comprehensive understanding of their collective impact on Construction Site Performance (CSP). Longitudinal studies evaluating the sustained effects of these strategies on construction projects over time would offer insights into their long-term efficacy. Comparative analyses across different regions, incorporation of diverse stakeholder perspectives, and assessments of newer technological advancements could enrich our understanding of material waste mitigation's contextual variations, stakeholder roles, and updated tools. Exploring regulatory influences, hybrid quantitative-qualitative studies, sustainability metrics integration, adoption challenges, and the economic and social dimensions of these strategies stand as potential areas for further investigation. Each of these avenues could amplify and refine our current understanding of material waste mitigation's implications for construction site performance.

## Data availability statement

The data supporting this study's findings can be obtained upon request from the corresponding author. Accessibility to the data is restricted to ensure the privacy of research participants.

## CRediT authorship contribution statement

**Mahdi Mohammed Abdullah Abkar:** Writing – original draft, Resources, Formal analysis, Data curation, Conceptualization. **Riduan Yunus:** Supervision, Project administration, Conceptualization. **Yaser Gamil:** Writing – review & editing, Funding acquisition, Formal analysis. **Mohammed Abdo Albaom:** Writing – review & editing, Methodology.

## Declaration of competing interest

The authors declare that they have no known competing financial interests or personal relationships that could have appeared to influence the work reported in this paper.
